# Prevalence, Etiology, and Antibiotic Resistance Patterns of Urinary Tract Infections in a Baghdad Hospital: Focus on Uropathogenic *E. coli* and Virulence Factors

**DOI:** 10.1155/ijm/9117996

**Published:** 2026-04-20

**Authors:** Huda Saad Salman, Hala Mohammed Majeed, Yusur Fadhil Shallal

**Affiliations:** ^1^ Department of Microbiology, Collage of Medicine, Ibn Sina University for Medical and Pharmaceutical Sciences, Baghdad, Iraq; ^2^ Department of Microbiology, Collage of Science, Al-Karkh University for Science, Baghdad, Iraq

**Keywords:** antibiotics, urinary tract infection, urine sample, uropathogens, virulence factors

## Abstract

**Background:**

Urinary tract infections (UTIs) remain one of the most common bacterial infections worldwide and represent a growing public health concern due to increasing antimicrobial resistance.

**Objective:**

A cross‐sectional observational study conducted at a hospital in Baghdad, Iraq, analyzed patients that attended for uropathogenic *Escherichia coli* (UPEC). It sought to estimate UPEC prevalence in patients, identify etiological agents of UPEC infections, assess UPEC antimicrobial susceptibility, and examine UPEC virulence factors.

**Methods:**

Between February and August 2024, there was a total of 182 midstream urine samples collected from patients clinically suspected of having a UTI. Bacteria were identified and antimicrobial susceptibility testing was performed via standard microbiological methods according to CLSI standards. Phenotypic assessments of biofilm formation, hemolysin production, and hemagglutination were performed on the *E. coli* isolates.

**Results:**

The study has shown that 95 of the samples (52.2%) had a positive culture for significant bacteriuria. *E. coli* was overwhelmingly the most commonly isolated organism at 63.2%. *Klebsiella* spp. and other nonhemolytic Gram‐negative bacteria were also isolated. A high level of resistance to both ampicillin and trimethoprim–sulfamethoxazole was found, whereas the highest level of susceptibility to imipenem and nitrofurantoin was found. A percentage of 71.7% of UPEC organisms had the ability to form a biofilm, 41.7% demonstrated the ability to lyse red blood cells (hemolysis), and 58.3% had the ability to agglutinate RBCs (hemagglutination). Biofilm formation and the ability to agglutinate RBCs were found to be significantly related to either antimicrobial resistance at the univariate level (*p* ≤ 0.05).

**Conclusion:**

These findings highlight the ongoing burden of antimicrobial resistance among uropathogens in Baghdad and emphasize the importance of continuous surveillance and rational antibiotic use.

## 1. Introduction

Urinary tract infections (UTIs) have the largest incidence of any type of bacterial infection globally, and they are an enormous public health burden for people of every age [1, 2]. Every day, USA’s millions of outpatient visits and hospitalizations to treat UTIs contribute to considerable healthcare costs and the disease itself [3]. Most UTI causative organisms are Gram‐negative, with *Escherichia coli* being the most commonly identified UTI pathogen, whether an acquired UTI occurs in the community or in the hospital [4–6].

Uropathogenic virus susceptibility has decreased as a result of the ever‐present threat from antimicrobial resistance, making treatment of these infections more difficult and limiting the drugs which can be used to treat them [7, 8]. Many patients with UTIs will be treated with widely used antibiotics such as ampicillin, trimethoprim–sulfamethoxazole, and the fluoroquinolone family of antibiotics due to their historical efficacy. There are now numerous reports from developing nations indicating that these antibiotics are often resistive [9–11], highlighting the importance of regional antimicrobial susceptibility surveillance to assist with selection decisions and improve clinical outcomes for patients [12].

Various virulence characteristics contribute to uropathogenic *E. coli’s* (UPEC) virulent potential in terms of how well it can colonize, persist, and infect the urinary tract [13]. These virulence traits include biofilm formation, hemolysin production, and hemagglutinating activity and play an important role in allowing UPEC strains to persist longer than they would without these virtues, thus providing increased antimicrobial resistance [14].

Undoubtedly, the resistance of UPEC to various antibiotics has increased significantly and represents a major threat to public health throughout the world. Regular surveillance of local distribution of UPEC strains is therefore required for monitoring their antibiotic resistance. Thus, the objectives of this study were to determine the prevalence and etiological agents of UTIs and to evaluate the antimicrobial susceptibility patterns and selected phenotypic virulence factors (biofilm formation, hemolytic activity, and hemagglutination) in UPEC isolated from patients presenting to the Private Nursing Hospital, Medical City Complex, Baghdad, Iraq, during the period from January through June 2024.

## 2. Materials and Methods

### 2.1. Study Design, Sampling Strategy, and Location

An observational, quantitative, cross‐sectional study was conducted at the Private Nursing Hospital at Medical City Complex, Baghdad, Iraq, from January to June 2024 with a consecutive sampling strategy of all patients diagnosed with UTI within the study period using average annual UTI case attendance at the hospital the previous year. The sample size (*n* = 182) was determined based on average number of cases per expected arrival date and was sufficiently large to calculate reliable prevalence estimates during the study period.

### 2.2. Inclusion and Exclusion Criteria

Patients of both sexes with different age groups presented with the abovementioned signs and symptoms. As for exclusion criteria, patients on antimicrobial therapy with missing information and patients with other microbial infections were excluded.

### 2.3. Urine Collection and Culture

Five mL of urine was collected from the mid‐urethra and incubated with brain and heart infusion broth at 37°C for 48 h. In addition, urine strips were placed on blood and MacConkey agar at 37°C for 48 h. Culture characterization and biological testing. Significant bacteriuria was defined as the presence of ≥ 10^5^ colony‐forming units (CFU)/mL of a single organism, according to standard microbiological diagnostic criteria. The VITEK2 system was used to diagnose all emerging bacterial isolates [15].

### 2.4. Antibiotic Susceptibility Testing (AST)

Antimicrobial sensitivity was evaluated through the disk diffusion technique. Eleven antibiotic disks (Biomaxima, Poland) were tested, including amikacin (30 μg), aztreonam (30 μg), azithromycin (15 μg), trimethoprim (5 μg), levofloxacin (5 μg), tetracycline (30 μg), cefotaxime (30 μg), chloramphenicol (30 μg), ciprofloxacin (5 μg), ceftriaxone (30 μg), and gentamicin (10 μg). Bacterial colonies were diluted in a nutrient broth (0.5 McFarland standard, ∼1.5 × 10^8^ CFU/mL) and standardized. The suspensions were then spread onto Mueller–Hinton agar plates (Oxoid). After placing the antibiotic disks, the plates were incubated at 37°C for 24 h under aerobic conditions. Interpretation of inhibition zone diameters was performed according to the Clinical and Laboratory Standards Institute (CLSI) guidelines. *E*. *coli* ATCC 25922 was used as a quality control strain for antimicrobial susceptibility testing [15].

### 2.5. Biofilm Formation

Biofilm production in UPEC and control strains was evaluated using Congo red agar. The medium contained brain heart infusion broth (37 g/L), sucrose (50 g/L), agar (10 g/L), and Congo red stain (0.8 g/L), which was added after autoclaving. Bacterial cultures were streaked onto the plates and incubated at 37°C for 24 h under aerobic conditions. Biofilm‐forming *E. coli* strains were identified by the development of black colonies exhibiting a dry, crystalline morphology [16].

### 2.6. Hemolysin Activity

The UPEC and control strains were cultured in peptone water and incubated at 37°C until achieving a turbidity equivalent to a 0.5‐McFarland standard. The bacterial suspensions were then streaked onto blood‐enriched agar plates containing 5% sheep blood and incubated at 37°C for 18–24 h. Hemolytic activity was assessed by the presence of β‐hemolysis, characterized by a transparent zone surrounding the bacterial colonies due to complete red blood cell lysis [17].

### 2.7. Hemagglutination Test

Bacterial hemagglutination was assessed using a slide agglutination assay following established protocols [18]. Freshly prepared 1% nutrient broth was inoculated with bacterial isolates and incubated at 37°C to promote fimbrial expression. After centrifugation, the pelleted bacteria were gently resuspended in the remaining broth to enhance adhesion exposure. A 3% erythrocyte suspension was prepared using human Group O blood, with red blood cells (RBCs) washed three times in physiological saline before final dilution. Equal volumes of the bacterial suspension and RBCs were mixed on a glass slide and allowed to react at ambient temperature for 10 min. Visible agglutination indicated a positive hemagglutination reaction.

### 2.8. Data Analysis

Data were analyzed using SPSS Version 26. Descriptive statistics were presented as frequencies and percentages. Associations between categorical variables were evaluated using Chi‐square or Fisher’s exact test where appropriate. A *p* value of < 0.05 was considered statistically significant.

### 2.9. Ethical Approval

This study was performed within the guidelines set forth by the Declaration of Helsinki and followed the ethical principles set forth by Ibn Sina University’s Scientific and Ethical Committee (Approval No: 5/1/2024. Adult subjects provided written informed consent; however, subjects less than 18 years of age had consent provided by their parent/legal guardian. Blood from humans that is used in hemagglutination testing was collected from healthy volunteers with their informed consent using procedures established by this institute.

## 3. Results

### 3.1. Analysis of Demographic Variables and Disease Profile

A total of 182 urine samples were collected from patients with clinical suspicion of UTI. Significant bacteriuria was detected in over half of the examined samples (Table 1).

**TABLE 1:** Prevalence of urinary tract infection.

Variable	Data
Total number of participants	182
Age (rang) (years)	0–89
Mean ± SD age	40 ± 23.9
Total number of females (%)	90 (49%)
Total number of males (%)	92 (51%)

### 3.2. Association of Infection With Age and Sex of the Participants

*E. coli* was identified as the predominant etiological agent, followed by *Klebsiella* spp. and other Gram‐negative bacteria (Table 2). Gram‐positive isolates were detected at lower frequencies than Gram‐negative organisms.

**TABLE 2:** Prevalence of UTI cases according to sex and age groups.

Age group	Negative cases positive cases				Total (%)
	Female (%)	Males (%)	Female (%)	Male (%)	
0–9	7 (15)	6 (10)	4 (9)	4 (12)	21 (12)
10–19	6 (13)	10 (17)	4 (9)	0 (0)	20 (11)
20–29	8 (17)	7 (12)	7 (16)	7 (21)	29 (16)
30–39	8 (17)	10 (17)	6 (14)	3 (9)	27 (15)
40–49	4 (9)	4 (7)	5 (12)	4 (12)	17 (9)
50–59	2 (4)	6 (10)	4 (9)	3 (9)	15 (8)
60–69	7 (15)	6 (10)	7 (16)	5 (15)	25 (14)
70–79	3 (6)	8 (14)	3 (7)	7 (21)	21 (12)
80–89	1 (4)	1 (2)	3 (7)	1 (3)	7 (5)
Total	47 (100)	58 (100)	43 (100)	34 (100)	182 (100)

### 3.3. Microbial Causative Agents of UTI

The distribution of UTI cases according to age group and sex is presented in Table 3. Although female patients represented a higher proportion of cases, statistical analysis did not demonstrate a significant association between sex and infection rate.

**TABLE 3:** Prevalence of uropathogens isolated from the positive cases.

Microbial causative agent	Genus and species	Number (%)
Fungal	*Candida* spp.	13 (17)
Bacterial	*E. coli*	28 (44)
	*Klebsiella* spp.	13 (17)
	*Pseudomonas* spp.	9 (12)
	*Enterobacter* spp.	8 (10)
	*Streptococcus* spp.	4 (5)
	*S. aureus*	2 (3)

### 3.4. AST

Table 4 clearly outlines the antimicrobial susceptibility profiles displayed by the isolated uropathogens. Many of the commonly prescribed antibiotics had significantly elevated rates of resistance, while carbapenems and nitrofurantoin exhibited comparable and high activities against numerous isolates of microorganisms.

**TABLE 4:** Antimicrobial susceptibility testing.

Antibiotics	Sensitive		Resistant	
	Frequency	Percentage (%)	Frequency	Percentage (%)
Amikacin	50	78	14	22
Azithromycin	40	63	24	36
Aztreonam	9	14	55	86
Cefotaxime	20	31	44	69
Ceftriaxone	35	55	29	45
Ciprofloxacin	46	72	18	28
Chloramphenicol	30	47	34	53
Gentamycin	24	38	40	62
Levofloxacin	38	59	26	41
Tetracycline	14	22	50	78
Trimethoprim	22	34	42	66

### 3.5. Virulence Factors of UPEC

The occurrence of various virulence factors, such as biofilm production, hmuY, and hemagglutination, can be seen among the different UPEC isolates (Table 5). Biofilm production was the most commonly identified virulence factor in this study.

**TABLE 5:** Expression of virulence factors of UPEC.

Virulence factors	UPEC (*n* = 28) (%)	Control (*n* = 12) (%)	*p* value
Biofilm formation	23 (77)	4 (20)	< 0.001^∗^
Beta‐hemolysis	6 (18.9)	2 (10)	0.122
Hemagglutinin	20 (70.2)	4 (20)	< 0.001^∗^

^∗^Statistically significant at *p* < 0.05.

At the univariate level of analysis, there was a statistically significant relationship between biofilm production and hemagglutination and antimicrobial resistance. No statistically significant relationship was seen between hemolysin production and antimicrobial resistance (Table 5).

## 4. Discussion

The present study aims to identify the predominant microbial etiological agents of UTI and their drug susceptibility patterns among patients. The findings showed an overall UTI prevalence rate of 42%, with bacterial infections accounting for 83% of the confirmed cases. This aligns with previous studies conducted in Iraq and the United Kingdom [19], which also reported a significantly higher prevalence of bacterial UTIs compared to those caused by fungal pathogens [20].

Within the study, females accounted for a slightly larger number of cases of UTIs (56% of all UTI cases) than males (44% of all UTI cases). This is consistent with past studies which have demonstrated a higher prevalence of UTIs in females [21, 22]. However, the statistical analysis performed within the present study did not indicate that there was a significant association between gender and the prevalence of UTI in the cohort studied; therefore, gender did not appear to be an independent predictor of having a UTI.

The increased susceptibility of females to acquiring UTIs has often been attributed to a variety of factors associated with females having different anatomies than males, such as having shorter urethras and being positioned close to the anus, physiological factors such as hormonal fluctuation associated with pregnancy and menopause, and behavioral factors such as engaging in sexual activities [23, 24]. Although these risk categories have been documented in the literature, they were not statistically validated by the current data collected in this cohort.

Age‐distribution patterns reveal two age‐specific groups demonstrated increased frequency of infections; specifically young adults (20–29 years) and older adults (60–69 years old). Similar bimodal distribution of infection has been documented previously [25, 26]. Younger adults could have increased risk of infection because of increased exposure to behaviorally associated risks, whereas older adults could have increased susceptibility to infection due to a variety of reasons including chronic illness (comorbidities), weakened immune system, urinary retention, prostatic enlargement, or neurogenic bladder. More analytical studies are needed in order to identify independent age‐associated risk factors.

UTIs are attributed to many bacterial causative agents, including Gram‐positive and Gram‐negative. The results showed that the members of Enterobacteriaceae caused more than 70% of the positive cases. The same findings were also demonstrated in other studies [27, 28], showing that members of this family were considered the primary cause of UTI in both community and nosocomial settings. The largest number of positive cases is associated with *E. coli*, due to its natural presence in the body and the presence of pathogenic elements. It allows its transition from the lower to the higher system. *Klebsiella* spp. is known for its capsular and high virulence factors, which facilitate the transmission of diseases. Similar results have been found in other studies [29, 30].

Regarding AST, our results show that amikacin, azithromycin, ceftriaxone, ciprofloxacin, and levofloxacin show higher sensitivity than others, whereas other drugs show high resistance levels. Our results agreed with those of a recent study carried out in Ethiopia in 2021, which reported that Gram‐negative uropathogens were highly sensitive to ciprofloxacin and resistant to tetracycline, trimethoprim, and cefotaxime [31]. UTIs represent a major global health concern, with *E. coli* being the primary causative agent affecting most individuals during their lifetime. The emergence of antimicrobial resistance in UPEC has escalated into a serious public health challenge. Current understanding of the relationship between UPEC’s antibiotic resistance profiles and its phenotypic/genotypic features remains limited, particularly in the Iraqi population. While some international studies have explored these associations [32], significant knowledge gaps persist regarding regional patterns of resistance and virulence. UPEC employs multiple pathogenic mechanisms to establish infections, with disease severity closely tied to the expression of specific virulence determinants [11]. Characterization of these factors and their interplay with antimicrobial resistance could provide critical insights into UTI progression and enable better management of potential complications, including pyelonephritis and impaired renal function [32]. Our investigation focused on three key virulence properties: biofilm production, hemagglutination capability, and hemolytic activity. Biofilm formation represents a critical virulence mechanism, offering bacterial pathogens’ dual protection against both host immune defenses and antimicrobial treatments. Our findings demonstrated significantly greater biofilm production in UPEC isolates (77%) compared to control strains (20%), aligning with previous reports identifying biofilm as a predominant virulence trait in uropathogenic bacteria. This enhanced capacity for biofilm formation likely contributes substantially to UPEC’s persistence in the urinary tract and its recalcitrance to therapy [33, 34]. Hemagglutinin expression plays a significant role in facilitating the adhesion of isolates to the uroepithelial surface, followed by biofilm formation and host colonization [35]. This is consistent with the high number of biofilm formations in UPEC isolates in our study.

Our investigation revealed that 18.9% of UPEC isolates exhibited hemolytic activity, while only 10% of control strains demonstrated this characteristic. These findings align with regional research from Iraq and adjacent nations [36]. The higher prevalence of hemolysin production among UPEC strains holds clinical significance, as this virulence factor has been strongly associated with pyelonephritis cases. This correlation suggests that hemolytic capability may contribute substantially to the disease progression and tissue invasion observed in upper UTIs caused by UPEC [37].

Clinically, the increased resistance to frequently used antibiotics used to empirically treat UTIs in this type of setting may create difficulties for providing adequate clinical care. In order to manage resistance rates and assist with evidence‐based prescribing and utilizing local antimicrobial stewardship programs and conducting routine surveillance, these results further indicate the need for local antimicrobial stewardship programs. From the viewpoint of public health, ongoing assessment of resistance patterns is vital to control the spread of multidrug‐resistant uropathogens in order to decrease healthcare costs and the quantity of unsuccessful treatment outcomes.

## 5. Conclusions

The study presents new epidemiological information regarding UTIs and antimicrobial resistance trends based on data collected from hospitals within Baghdad, Iraq. The findings confirm that *E. coli* is the most common uropathogen‐causing UTIs; however, it is also highly resistant to many first‐line antimicrobials commonly used for treating UTIs. Several virulence factors associated with pathogenicity have been identified in *E. coli* strains isolated from patients with UTIs; these include the ability to form biofilms and the ability to hemagglutinate. The establishment of a consistent surveillance system and development of ambulatory care antimicrobial management protocols are necessary to improve the treatment of complex UTIs and minimize the transmission of antibiotic‐resistant UPEC or other bacterial strains resistant to first‐line therapeutic agents.

## Author Contributions

All authors have contributed substantially to this research, with individual contributions detailed below: Huda Saad Salman and Yusur Fadhil Shallal: study conceptualization and design, overall project supervision, statistical analysis of data, and critical revision of the final manuscript. Huda Saad Salman: sample collection, conducting laboratory experiments, and design and preparation of tables and figures. Hala Mohammed Majeed: comprehensive literature review, preparation of the initial manuscript draft, and formatting the manuscript in accordance with journal guidelines.

## Funding

The authors declare that no funds, grants, or other support were received during the preparation of this manuscript.

## Disclosure

All authors have read and approved the final version of the manuscript and take full responsibility for its content.

## Conflicts of Interest

The authors declare no conflicts of interest.

## Data Availability

The data that support the findings of this study are available from the corresponding author upon reasonable request.
